# Precision at the Margin: Innovations and Challenges in Intraoperative Molecular Imaging for Thoracic Surgery

**DOI:** 10.3390/jcm15124493

**Published:** 2026-06-10

**Authors:** Emily P. Rabinovich, Linda W. Martin

**Affiliations:** 1 Department of Surgery, University of Virginia Health System, Charlottesville, VA 22908, USA; epr3wxp@uvahealth.org; 2 Division of Cardiothoracic Surgery, Department of Surgery, University of Virginia Health System, Charlottesville, VA 22908, USA

**Keywords:** intraoperative molecular imaging, thoracic oncology, lung cancer, fluorescence-guided surgery

## Abstract

Tumor localization during pulmonary surgery has become increasingly challenging with the earlier detection of smaller and smaller lung nodules. Concomitantly, minimally invasive surgical (MIS) techniques have been increasingly adopted within the field of thoracic surgical oncology. Surgeons face growing challenges not only with locating these small tumors, but also with immediate margin assessment, reduced tactile feedback, and nodal assessment. Intraoperative molecular imaging (IMI) has emerged as a promising adjunct to address these challenges by enabling real-time visualization of malignant tissue during pulmonary resection. In its current form, IMI integrates systemically administered, tumor-targeting near-infrared fluorophores with fluorescence-capable imaging platforms to enhance intraoperative decision-making. Early clinical experiences in thoracic surgery suggest particular utility in the localization of small or nonpalpable pulmonary nodules and for improved margin assessment during MIS. Despite encouraging preliminary data, widespread adoption of IMI remains limited by biologic variability in target expression, optical depth constraints, false-positive fluorescence in inflammatory tissue, and challenges in workflow integration. Applications for nodal evaluation, staging, and longer-term oncologic outcome improvement remain investigational. Addressing these multifaceted barriers will be essential for the translation of IMI from a promising, experimental adjunct to a more broadly implementable surgical technology. This work summarizes the current state of IMI in thoracic surgical oncology, highlighting key translational studies, established and emerging clinical applications, and critical limitations within the current landscape. The authors also outline future directions for the field, including quantitative fluorescence interpretation, standardized reporting, and outcomes-driven clinical trials evaluating margin adequacy, recurrence, staging impact, and cost-effectiveness to support widespread evidence-based implementation.

## 1. Introduction

As long as cancer surgery has existed, surgeons have relied on two tools to locate tumors during an operation: sight and touch. Minimally invasive (MIS) thoracic surgery increasingly relies on visual rather than tactile cues, introducing novel challenges in tumor localization, margin assessment, and nodal evaluation for patients with lung malignancies. Intraoperative molecular imaging (IMI) has emerged as “a new tool in the surgeon’s armamentarium,” as described by Singal and colleagues, leveraging tumor-specific fluorescence to enable real-time visualization of malignant lesions during surgical resection [1]. In practical terms, IMI functions as an intraoperative imaging adjunct, conceptually analogous to an “intraoperative PET scan,” in that it helps surgeons identify otherwise occult malignant tissue intra-operatively, rather than pre-operatively.

This new technology addresses several critical surgical challenges in MIS lung cancer resections: the localization of nonpalpable lesions, margin assessment at the time of surgery, the detection of occult synchronous lesions, and identification of residual disease [2,3]. These challenges have become more pronounced as thoracic oncology has shifted toward widespread adoption of video-assisted thoracoscopic and robotic approaches, increased detection of small and non-solid pulmonary nodules, such as ground glass opacities, with improved screening, and parenchyma-sparing resections. While preoperative localization techniques, intraoperative palpation, and frozen-section analysis are integral to surgical decision-making, each is associated with notable limitations, particularly during minimally invasive procedures, for deep or nonpalpable lesions, and for real-time margin assessment [4,5]. Surgical resection continues to serve as the cornerstone of treatment for solid tumors, with complete (R0) resections remaining the most powerful predictor of long-term survival across tumor types [6,7]. Consequently, there remains a critical unmet need for intraoperative tools that provide biologically informed guidance beyond conventional visual assessment. Clinically, the appeal of IMI lies in its ability to assist surgeons in localizing tumors without additional invasive localization procedures, identifying otherwise unrecognized nodules during the index operation, and providing immediate information when a margin appears close or involved.

IMI technology addresses this gap by utilizing tumor-associated molecular targets, coupled to near-infrared fluorophores, to enable real-time visualization of malignant tissue during pulmonary resection [8,9]. Recent clinical applications of these small-molecule targeted agents have demonstrated the feasibility of intravenous administration, favorable safety profiles, and successful intraoperative detection of both primary lung tumors and occult disease using commercially available imaging platforms [2,3,10,11]. These advances highlight IMI as a feasible technology that may be integrated into existing operative workflows with the potential to improve intraoperative decision-making and oncologic precision. Importantly, the ability to improve lesion detection and intraoperative decision-making at the time of the initial operation allows IMI to reduce the likelihood that these missed lesions or inadequate margins will necessitate additional procedures or reoperation.

Established alternatives for pulmonary nodule localization, including bronchoscopic marking and image-guided percutaneous localization by interventional radiology, remain important tools in thoracic surgical oncology [12]. However, these strategies often require separate preoperative procedures and anesthesia events, added cost, interdepartmental coordination, and scheduling complexity, as well as an additional invasive procedure before definitive resection [13,14]. In this context, IMI provides the potential to integrate lesion localization directly into the operation itself during the same anesthesia event.

This work reviews the current state of IMI in thoracic surgery, with particular emphasis on its impact on tumor localization and margin assessment, as well as potential applications in nodal evaluation. The authors highlight key clinical trials evaluating the efficacy of these technologies, discuss technical and practical barriers to widespread adoption, and outline future directions to establish IMI as an evidence-based adjunct to current practice patterns in precision thoracic oncology.

While early preclinical and clinical data remain promising, IMI represents a potential adjunct to conventional localization and pathologic margin assessment and may have a role in systematic nodal evaluation. The current evidence supports its use in selective high-yield intraoperative scenarios, such as in the case of localization of small, nonpalpable, or visually occult pulmonary lesions, identification of unexpected malignant disease at the time of an index operation, and adjunctive assessment of close parenchymal margins during MIS. The central question for the next phase of thoracic IMI is whether these fluorescence-guided intraoperative decision-making techniques can reproducibly improve clinically meaningful surgical and oncologic outcomes beyond tumor visualization alone.

## 2. Principles of Intraoperative Molecular Imaging

Intraoperative molecular imaging relies on the selective accumulation of fluorescent contrast agents in malignant tissue or the extracellular matrix, enabling real-time optical visualization during surgical resection. Unlike conventional white-light imaging utilized during traditional MIS techniques, which relies exclusively on physical features, IMI aims to render tumor tissue visible at the time of operative resection regardless of density or size. This becomes especially relevant in thoracic oncology, where MIS approaches, small lesion sizes, and visually occult tumor morphologies may often limit tactile and visual cues during resection [15].

Most clinically translated IMI strategies in thoracic surgery implement tumor-associated molecular targets coupled to near-infrared (NIR) fluorophores, such as pafolacianine and abenacianine [2,3,16]. The NIR spectrum offers favorable tissue penetration and reduced background autofluorescence relative to visible wavelengths. This spectrum allows for intraoperative detection of fluorescence signals within the lung parenchyma, which contains abundant collagen-rich connective tissue that otherwise generates significant autofluorescence with visible-light agents [6,17]. Small-molecule targeting approaches such as pafolacianine and abenacianine have demonstrated particular promise in IMI, given their rapid pharmacokinetics, same-day administration feasibility, and compatibility with standard perioperative workflows [2,3]. This is particularly relevant in thoracic surgical oncology, where minimizing additional preoperative visits and workflow complexity is desirable, especially for patients who require multidisciplinary care. Alternative approaches using monoclonal antibody fragments such as SGM-101 have demonstrated higher binding affinity and specificity, with tradeoffs in tumor accumulation and prolonged circulation, often requiring administration between one and five days before surgery [15,18]. Additional IMI platforms described in the literature are summarized in Table 1.

Since the clinical performance of IMI depends on both the available fluorophore and the imaging system available, these platforms are not interchangeable. Dose, timing of administration, wavelength, tumor-to-background ratio, target expression, measurable lesion depth, and camera sensitivity all influence whether a fluorescent signal is visible intraoperatively. As a result, the above-reported performance metrics across agents should be interpreted cautiously, particularly in cases where studies differ in tumor type and lesion depth.

As a whole, IMI should be conceptualized as an adjunct technology within perioperative workflows. Effective implementation of these technologies requires the systemic administration of molecularly targeted fluorophores, imaging, or visualization hardware capable of detecting and displaying fluorescence in real time, and a surgical workflow that integrates these imaging strategies without disrupting operative efficiency during a patient’s index operation.

## 3. Current Clinical Applications in Thoracic Surgery

### 3.1. Tumor Localization

**Figure 1:**
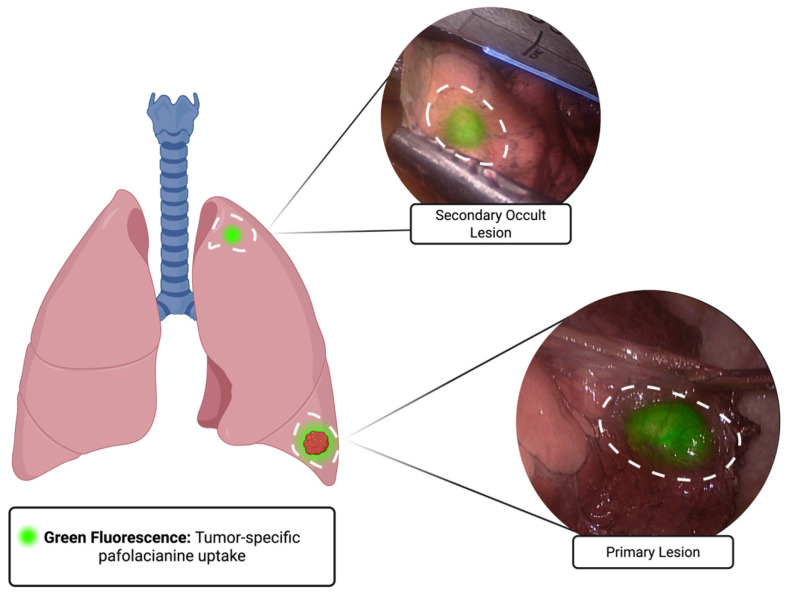
Schematic representation of two pulmonary lesions exhibiting tumor-specific fluorescence. Insets show corresponding intraoperative thoracoscopic views of the primary lesion in the left lower lobe and secondary occult lesion in the left upper lobe under fluorescence imaging, with dashed outlines highlighting areas of increased uptake relative to surrounding lung parenchyma. Green fluorescence denotes accumulation of pafolacianine, enabling enhanced intraoperative identification.

Current thoracic applications of IMI should be interpreted in the context of available clinical evidence. The leading and most established application of IMI in thoracic surgery has been intraoperative tumor localization, which is particularly relevant for small, deep, or nonpalpable pulmonary nodules. Fluorescence guidance has been shown to aid in identifying lesions that are occult on pleural inspection or incompletely localized by preoperative techniques. The ELUCIDATE trial, a phase three, 12-center trial involving 112 patients with suspected or biopsy-proven lung cancer scheduled for sublobar pulmonary resection, utilized the most clinically advanced IMI to date, pafolacianine, and identified synchronous malignant lesions outside the planned field, accounting for over 70% of fluorescence-identified lesions [3]. Follow-up studies branching from the ELUCIDATE trial demonstrated significant improvement in the identification of primary and occult malignant lesions when combined with standard practice compared with standard practice alone (*p* < 0.001) [19]. Studies evaluating abenacianine demonstrated efficacy in localizing lesions not found by standard surgical methods, providing broad histologic applicability across adenocarcinoma, squamous cell carcinoma, neuroendocrine tumors, and metastatic lesions [2,40]. These early clinical data highlight a key benefit of these technologies: the facilitated detection of additional malignant lesions not identified on preoperative imaging, raising the possibility of a more comprehensive oncologic resection (Figure 1). Importantly, these studies represent improvements in intraoperative detection and additions to standard surgical decision-making rather than global improvements in recurrence or survival. Therefore, current evidence supports IMI as a useful adjunct for selective localization challenges, rather than as a replacement for standard pathologic confirmation.

### 3.2. Margin Assessment

IMI has also been explored as an adjunct for intraoperative margin assessment during wedge resection and anatomic segmentectomy. Positive surgical margins occur in approximately 2.8–4.4% of lung cancer resections and are associated with significantly worse survival (5-year overall survival 34.5–44% versus 57–70% for negative margins) [5,41,42]. Fluorescence signal at or near the resection margin at the time of operative resection may provide real-time adjunctive feedback regarding the adequacy of parenchymal clearance, particularly in cases where gross margins are difficult to assess visually. In clinical terms, this raises the possibility that IMI may be able to alert the surgeon when the operative resection margin is closer than anticipated before the operation is completed, when additional resection may still be feasible. In a 2021 phase II multicenter trial, IMI revealed positive margins in 9% of samples that surgeons initially thought were negative using conventional white-light visualization [10]. In the ELUCIDATE trial, IMI identified close margins of less than 10 mm in approximately 38% of patients undergoing sublobar resection [3]. While IMI does not replace histopathologic confirmation, it complements anatomic margin assessment in MIS approaches.

### 3.3. Emerging Applications in Nodal Evaluation

The role of IMI in nodal evaluation remains investigational but conceptually compelling. Tumor-targeted fluorescence has the potential to highlight involved lymph nodes intraoperatively, while alternative approaches using non-targeted fluorophores such as ICG may assist with lymphatic mapping [43]. At present, however, evidence supporting routine fluorescence-guided nodal staging in thoracic oncology is limited, and fluorescence-positive nodes cannot be assumed to represent pathologic nodal disease. To date, there is a paucity of outcome-driven data supporting routine nodal imaging, but ongoing translational efforts suggest that IMI may help augment systematic nodal dissection [44]. Routine clinical adoption of nodal IMI will require further study.

## 4. Limitations and Barriers to Widespread Adoption

A central limitation of tumor-targeted fluorescence is biological variability. Target expression can vary by histologic subtype, tumor differentiation, and microenvironmental factors, producing heterogeneous fluorescence and false negatives [16,19,45]. Tumor-targeted fluorescence depends highly on adequate target expression, vascular delivery, tracer retention, and lesion proximity to the parietal pleura. Consequently, false-negative fluorescence may occur when the target is absent or heterogeneously expressed, when the lesion is too deep for sufficient transmission, or when the surrounding lung parenchyma attenuates signal [8,17]. False-negative fluorescence has been shown to occur in mucinous adenocarcinomas, heavy smokers with over 30 pack-years due to soot deposition and parenchymal inflammation, and tumors greater than 1.4 cm from the pleural surface [16,46]. These studies also demonstrate that non-malignant fluorescence may occur through inflammation or reactive cells surrounding some targets. As a result, it is essential to contextualize the findings, particularly when IMI identifies additional possible lesions.

These technologies have significant limitations with regard to depth and optical constraints. Even within the near-infrared spectrum, light scattering and absorption impose fundamental depth limitations, particularly with subsurface lesions [8,17]. Fluorescence performance is therefore best at exposed or near-surface lesions and may be reduced for deep parenchymal nodules unless dissection exposes tissue planes sufficiently for detection. Though depth detection is routinely reported, depth should not be interpreted solely as a fixed property of a fluorophore, as it varies depending on wavelength, signal intensity, tissue composition, tumor-to-background ratio, camera sensitivity, and lesion location [17]. Although some novel NIR-II imaging shows promise for deeper penetration, standard NIR imaging has reported a depth limitation of 5–10 mm, with occasional detection up to 1.4 cm under favorable conditions [8,47,48]. Ongoing areas of investigation are evaluating ways to overcome this—for example, use of higher dosing of flourophores, alterations in timing of administration, different optical equipment or technique.

In addition to the above considerations, IMI introduces steps that must be harmonized with perioperative thoracic workflows, integrating preoperative agent administration, intraoperative camera setup, and standardized specimen imaging. Machine learning-enhanced IMI analyses have been shown to produce results in less than two minutes, compared with an average of 34 min for frozen-section analysis, potentially reducing the clinical and financial impact of anesthesia time and operating room utilization [46]. Adoption of these technologies at scale will depend on whether IMI demonstrates significant downstream value that offsets incremental costs, such as reduced conversions, fewer re-resections, improved margin adequacy in limited resections, or improved detection of clinically meaningful synchronous lesions. Clinical impact data support the value proposition of these technologies. The Phase III ELUCIDATE trial reported a rate of 53% of patients in whom IMI identified at least one clinically significant event that altered surgical management, exceeding the prespecified threshold of 10% (*p* < 0.0001) [3]. While many IMI studies have emphasized feasibility and clinical benefit, there remains a paucity of literature on the economic considerations and robust cost-effectiveness data, which will be critical for the widespread adoption of these technologies.

Cost and implementation barriers are important considerations, yet thus far, there is a paucity of information on this in thoracic IMI literature. The economic burden of IMI includes the fluorophore itself, pharmacy preparation and vehicle, infusion strategy and logistics, nursing and anesthesia coordination, intraoperative imaging time, and access to compatible NIR thoracoscopic and robotic platforms [29]. These costs differ substantially depending on the current availability of technology within a single institution and reimbursement pathways for these agents. Existing technologies such as navigational bronchoscopic or image-guided injection also come with significant costs, possible procedure-related complications and multiple anesthetic events. Thus, future studies should evaluate not only lesion detection and diagnostic accuracy, but also downstream outcomes such as avoidance of separate localization procedures, re-resection rates, operating room time, and total costs associated with the use of these fluorophores or any other localization strategy.

To transition these technologies into a scalable clinical tool, thoracic IMI will require standardization of fluorescence strategy, implementation across different camera systems and institutions, and signal interpretation. This includes calibration practices, reporting standards for tumor-to-background ratio descriptions, and documentation of operative workflows that enable cross-center comparability [10,46,49]. Future studies should increasingly prioritize reporting endpoints aligned with adoption to current practice, such as margin adequacy and local recurrence after limited resections, frequency and clinical significance of synchronous lesion detection, impact on staging, reintervention, and cost-effectiveness. These endpoints will be essential for defining where IMI offers reproducible value for thoracic oncology surgical patients and moving the needle past mere technical feasibility.

## 5. Conclusions

Lung nodule localization is a growing challenge with earlier cancer detection and increased adoption of minimally invasive surgery. Various strategies have been tried over the years, but one that holds great promise is intraoperative molecular imaging. It has evolved from a proof-of-concept fluorescence visualization strategy to a practical surgical adjunct that has demonstrated significant capability for augmenting intraoperative decision-making in thoracic oncologic surgery, particularly as surgeons increasingly manage smaller, less palpable lesions through minimally invasive approaches. The current evidence supports its greatest clinical utility in selected cases requiring optimized tumor localization or immediate margin assessment, while features such as nodal evaluation and broader staging applications remain investigational. Multi-institutional clinical trials and analyses have reported meaningful outcomes with IMI utilization. As a whole, the clinical promise of IMI lies in its ability to help surgeons localize tumors, identify additional malignant lesions that would otherwise not be visible to the naked eye, and assess margin adequacy during a single operation, potentially reducing the need for additional invasive localization or future reintervention. Prior to widespread implementation of IMI as a standard component of thoracic oncologic resection, the field must address the impact of biologic heterogeneity, optical depth limitations, false-positive and false-negative interpretation, platform standardization, workflow integration, and cost-effectiveness. Future stages of progress for these technologies will increasingly be defined by standardization, quantitative interpretation of fluorescence signals, and outcomes-driven analyses, thereby integrating IMI as a useful adjunct to precision thoracic oncology.

## Figures and Tables

**Table 1:** Representative Fluorophores and Platforms Relevant to Intraoperative Molecular Imaging in Thoracic Surgery.

Target/Strategy	Example Agent/Trial Identifier	Targeting Class	Development/ Regulatory Status	Potential Thoracic Applications	Cost/Dose/Timing	Performance and Depth	Safety/Key Limitations	Refs.
Tumor-associated receptor targeting	Pafolacianine (OTL38)/NCT04241315	Small-molecule, receptor-targeted (folate receptor-α/β)	Based on the current FDA label, pafolacianine has two approved indications: identification of malignant and nonmalignant pulmonary lesions and intraoperative identification of malignant lesions in adult patients with ovarian cancer (2021)	Tumor localization, margin assessment (identification of margins ≤ 10 mm in 38% of sublobar resections), occult synchronous lesion detection, may be administered 1–24 h preoperatively	Requires FDA-cleared NIR imaging system. 0.025 mg/kg administered as a single IV infusion over 60 min, 1 to 24 h before surgery.	Incremental lesion localization in patients where lesion was otherwise nonpalpable/not visualized, 19%; synchronous malignant lesion detection, 8%; detected depth of lesion 0 to 38 mm from lung surface.	No drug-related serious adverse events. Nausea, vomiting, abdominal pain, flushing, hypersensitivity. Target heterogeneity, limited depth penetration.	[3,16,19,20,21,22]
Non-targeted perfusion imaging	Indocyanine green (ICG)/NCT02611245	Non-specific dye	Widely available. FDA approved for multiple indications, including vessel/perfusion visualization, lymphatic mapping). High-dose preoperative systemic ICG for pulmonary nodule localization remains off-label/investigational.	Intersegmental plane identification, nodule localization, perfusion, lymphatic mapping. Higher-dose ICG has also been investigated for tumor localization via the enhanced permeability and retention effect, although this remains non-specific.	Low-cost, widely available. Compatible with many existing imaging systems. Published, early-phase feasibility data using 5 mg/kg IV slow infusion administered 24 h before surgery.	Reported sensitivity for tumor localization approximately 89%, PPV 92.6%, limited tissue penetration depth, unable to distinguish tumor from inflammation. Reported depth detection in lung tissue reported for lesions up to 1.3–1.4 cm from the pleural surface.	Rare anaphylactic/urticarial reactions.	[23,24,25,26,27,28,29,30,31]
Growth factor targeting	Bevacizumab–IRDye800CW	Antibody–dye conjugate	Early-phase (I/II) clinical trials for multiple cancer types	Tumor visualization, research applications, limited thoracic-specific data, evaluated primarily in peritoneal carcinomatosis, breast cancer, soft-tissue sarcomas, pancreatic cancers	Investigational/higher cost. Fixed doses such as 4.5, 10, or 25 mg, generally 2–4 days before surgery in early-phase studies.	Performance data unavailable for thoracic applications. Limited depth penetration.	No reported allergic or anaphylactic reactions or tracer-related adverse events. Long lead time (approximately 3 days), workflow complexity.	[8,32]
EGFR targeting	ABY-029	Affibody–dye conjugate	Early human studies	Preclinical and ex vivo studies in lung cancer demonstrating 85.7% sensitivity and 100% specificity for tumor identification, NSCLC visualization	Specific per-dose cost data unavailable; reviews have noted that small-molecule tracers such as these can be routinely produced at lower cost compared to antibody-based tracers. First-in-human studies used 30, 90, or 171 nmol 1–3 h before surgery.	Formal performance unavailable for human trials. Preclinical glioma trials report 91% diagnostic accuracy in EGFR + tumors.	No ABY-029-related adverse events reported in first-in-human sarcoma trial across all doses. Limited clinical data in thoracic oncology, stability testing limitations.	[33,34,35]
CEA targeting	SGM-101/NCT04315467, NCT04737213	Antibody–dye conjugate	Phase II/III (non-thoracic primaries), not FDA approved	Selected thoracic tumors/metastases	Cost data unavailable, production costs anticipated to be higher than small-molecule tracers. 10 mg approximately 2–4 days before surgery.	In vivo, 31% tumors demonstrated positive fluorescence when examining colorectal lung metastases. Tumor-to-background ratio of 1.00 in vivo.	No treatment-related adverse events. Tumor-specific expression required, requires 1–5 days of preoperative administration, slower pharmacokinetics compared with small molecule	[5,16,18,36,37]
Activatable probes	Protease-activated fluorophores, abenacianine (VGT-309)/ACTRN12618001472257, ACTRN12621000301864, NCT04563117, NCT05640986	Enzyme-responsive	Preclinical/early translational, Phase II multicenter trials for abenacianine	Improved specificity at margins, broad histologic applicability (adenocarcinoma, squamous, neuroendocrine)	Cost data unavailable, as small-molecule synthetic probe anticipated to be lower cost than other antibody-based tracers. Studied at 0.05, 0.16, 0.32 mg/kg; 2–6 h or 12–36 h preop; later studies selected 0.32 mg/kg 12–36 h prior to surgery.	Lesion detection rates ranging from 73–86% with dosing at 0.32 mg/kg at least 2–6 h prior to surgery. At least 42.5% of patients had at least one clinically significant event, defined as nodules not found by standard surgical methods. Tumor-to-background ratio of 2.15–7.18.	Phase 1 trials, no serious adverse events reported. One suspected unexpected adverse reaction of liver transaminitis, resolved within 30 days. No other reported infusion or drug-related events. Regulatory and interpretive complexity, potential for non-specific activation by inflammatory proteases, limited tissue penetration	[2,16,38,39]

## Data Availability

Not applicable.

## Abbreviations

The following abbreviations are used in this manuscript:

IMI	Intraoperative molecular imaging
MIS	Minimally invasive surgery
NIR	Near-infrared
ICG	Indocyanine green
